# Optimizing survival in Russell’s viper bite cases in low-resource setting: two case reports

**DOI:** 10.1186/s13256-024-04354-0

**Published:** 2024-02-15

**Authors:** Mohiuddin Sharif, Mohammad Robed Amin, Anindita Das Barshan, Mohammad Jahid Hasan, M. A. Faiz

**Affiliations:** 1 Dhaka Medical College Hospital, Dhaka, 1100 Bangladesh; 2 Noncommunicable Disease, Directorate General of Health Services, Mohakhali, Dhaka, 1212 Bangladesh; 3 Tropical Disease and Health Research Center, Dhaka, 1100 Bangladesh; 4 Pi Research Development Center, Dhaka, 1100 Bangladesh; 5 Chittagong Medical College & Hospital, Chittagong, 4203 Bangladesh; 6 Director General of Health Services, Mohakhali, Dhaka, 1212 Bangladesh

**Keywords:** Russell’s viper, Snake bite, Anti-snake venom, Health system delay, Low resource country

## Abstract

**Introduction:**

Snakebite envenomation poses a significant health risk, particularly in low-resource settings where access to proper treatment is limited.

**Case presentation:**

This study reports two cases of Russell's viper bites in rural Bangladesh, involving 48 and 35-year-old Bangladesh males, respectively, and highlights the difficulties in providing adequate medical care and in treating any complications that may arise. Both cases involved delayed access to healthcare, initial visit to traditional healers, and the development of severe complications such as coagulopathy, renal failure. After the intervention both cases survived which is scarce in low resource settings.

**Conclusion:**

The cases underscore the importance of early recognition, appropriate management, and improved healthcare infrastructure to optimize survival outcomes in snakebite cases in resource-limited settings. These cases will contribute valuable insights to the field of snakebite management and provide guidance for improving survival rates and outcomes among snakebite victims in Bangladesh.

## Introduction

Snakebite envenomation was officially classified as Category A Neglected Tropical Disease by the World Health Organization (WHO) on June 9th, 2017 [1]. It is a common medical emergency, frequently encountered in many countries, particularly in rural settings where anti-snake venom may not be easily accessible. Russell’s viper (*Daboia russelii*) is one of the most venomous snakes in the world and is responsible for a significant number of deaths and complications in Southeast Asia. Given its agricultural landscape and rural regions, Bangladesh is particularly vulnerable to snakebite incidents. Therefore, the prevalence of snakebite incidents, including those caused by Russell’s vipers, is notably heightened within the country. Tragically, between the years 2013 and 2016, this heightened risk led to the unfortunate loss of 20 precious lives in Rajshahi [2]. There have been several case reports illustrating different types of complications following Russell’s viper bites in Bangladesh. Here, we present a case series of two patients who survived multiple complications following a Russell’s viper snake bite. These two clinical cases illustrate the diverse challenges faced in delivering prompt medical treatment, in addition to presenting a range of complications as clinical features. Both cases are marked by delayed access to medical treatment, initial reliance on traditional healers (Ojhas), and subsequent severe complications. These cases provide an opportunity to discuss the public health challenges of snakebites, the common clinical features, and the need for improved snakebite management in rural areas of Bangladesh.

### Case report 1

**Fig. 1:**
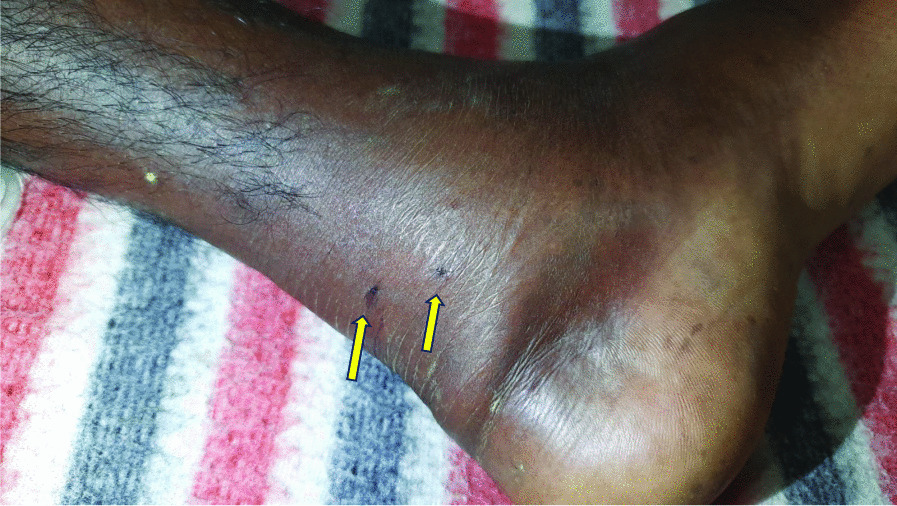
Bite of the Russel’s viper on the left leg (identified by yellow arrows)

A 48-year-old Bangladesh male farmer presented to the emergency department of Dhaka Medical College Hospital, a tertiary hospital in Bangladesh, approximately 18 h following a snakebite. He reported that he was laboring in a banana field when he suddenly felt sharp pain in his left leg (Fig. 1). He turned around and saw a snake slithering away. His fellow workers in the field rushed to his location, secured the snake in a large bowl, and immediately drove him to the nearest traditional healer (ojha), which is almost 3 km away from his event place. He applied some Green Chilies on the wound and recited some of his spells to negate the effects of the venom. After 90 min, the traditional healer confidently determined that the snakebite was from a nonvenomous and harmless snake, advising the patient to go back home. Unfortunately, upon arriving home, the patient's health worsened, leading his family to seek the help of another traditional healer located 12 km away. Like the first healer, the second healer employed various techniques to alleviate the patient's discomfort and affirmed that the snake in question posed no danger and was devoid of venom. Nevertheless, following consecutive treatments by two traditional healers for a duration of nearly 10 h, the patient's condition took a turn for the worse. This was evidenced by the emergence of new symptoms such as swelling in the affected leg and excruciating pain specifically localized in the bite site. The patient was promptly transferred to the closest upazilla health complex, where attending physicians thoroughly assessed his condition and determined that further specialized care was required. Then they were referred to Dhaka Medical College Hospital for more comprehensive management. Regrettably, during the patient's transit from the location of the snakebite to referred hospital, no medical intervention or treatment was administered. When first seen in the emergency room of this hospital, he was found to be extremely scared; however, he was conscious, oriented to time, place, and person. He complained about painful left leg and chest pain, as well as low urine output. On examination, his pulse rate was 112 beats per minute, blood pressure was 90/60 mmHg, and his respiratory rate was 24 breaths per minute, temp-98. He had two fang marks on his left lower leg, which were swollen and agonizingly painful. There was no evidence of any systemic allergic reaction. Clinically, cardiovascular, central nervous system, and respiratory system examinations had normal findings.

Initially, a bedside 20WBCT was performed which showed positive. The laboratory tests revealed a hemoglobin level of 14.1 g/dl, a total leukocyte count of 25,800/mm^3^ with a normal differential count, and a platelet count of 71,000/mm^3^. His peripheral blood film showed a fair number of target cells and echinocytes and evidence of microangiopathic hemolytic anemia, RBS-9 mmol/L. However, his coagulation profile showed a prolonged activated partial thromboplastin time (aPTT) of 25.8 s, an elevated prothrombin time (PT) of 25 s, and an INR of 2.16. The fibrinogen degradation product (2.6 mg/L) and D-dimer were positive (> 10 mcg/ml). S. Bilirubin 1.7 mg/dl (Direct-1.50 mg/dl, Indirect-0.20 mg/dl); AST 57 U/L. Urine examination revealed microscopic hematuria without hemoglobinuria. His renal function started deteriorating the day he was admitted, as evidenced by creatine kinase (804 U/L) mg/dl), blood urea of 81 mg/dl, and serum creatinine of 3.77 mg/dl. Na-133 mmol/l, K-6 mmol/L; Cl-105 mmol/L; serum albumin 3 gm/dl; corrected calcium 8.4 mg/dl. Baseline ECG-normal; CXR- normal. On the second day after admission, the investigation profile revealed bilateral pleural effusion on CXR and ascites in the abdomen on USG. However, dialysis was commenced (at day 2) due to poor renal output, acute hemolysis (decreased Hb 5 gm/dl within 24 h) and persistent increases in serum creatinine and serum urea. Due to worsening of the clinical condition, the patient was immediately shifted to the ICU (day 6).

**Fig. 2:**
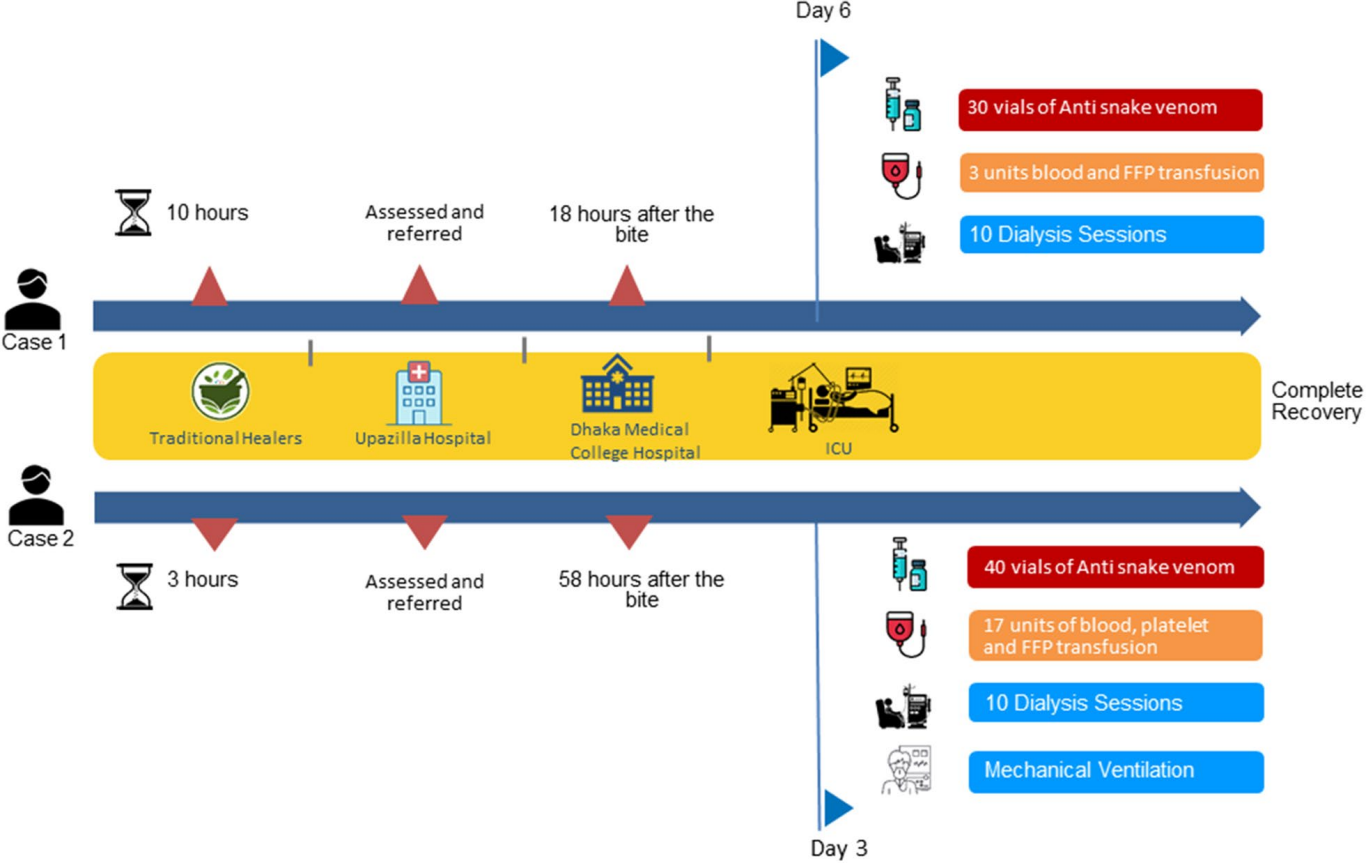
Case summary of two surviving cases following Russell’s viper bite

The patients received a total of 3 doses (30 vials) of antivenom along with intravenous fluids, analgesics, tetanus toxoid and immunoglobulin and third-generation cephalosporin. The patient also received 1 unit of FFP, 2 units of whole blood, and 10 sessions of dialysis. After one week of ICU stay, he was discharged from the hospital with no residual disability. During discharge, all of his blood and other parameters were within physiological limits. An ECHO was performed and revealed nothing significant. Summarized information are available at Fig. 2.

#### Outcome of the patients

The patient has survived without experiencing any disabilities. He regularly visits Rajshahi Medical College Hospital every two months for check-ups and maintains communication with the medical team through telephone calls. Additionally, any investigation reports and complaints are conveniently shared with the medical team via WhatsApp.

#### Outcome of the snake

The snake was brought by the relative of the patient. The snake was alive and transferred to the representative of the “Deep Ecology and Snake Conservation Foundation”, an organization that saves the snake in the natural environment. Later, the snake was left free to the natural habitat.

### Case 2

**Fig. 3:**
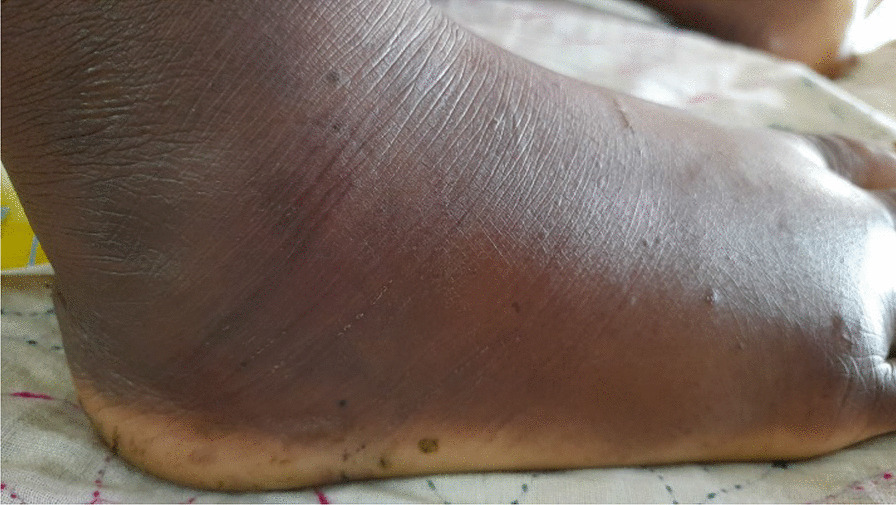
A site of snake bite and swelling of the foot (60 h after the bite)

A 35-year-old Bangladesh male businessman presented to Dhaka Medical College Hospital 58 h after a Russell’s viper bite while walking in a paddy field. The bite occurred at approximately 6.40 pm and resulted in a clear fang mark and bleeding from his leg. Then, he was quickly taken to a traditional healer who applied a tight round band around his right great toe and made bleeding using throne of flower in name of expulsion of toxin. Due to the absence of any noticeable improvement over time, the patient was transferred to the upazilla health complex within a few hours. Throughout this duration, the swelling in the patient's leg persisted, accompanied by continuous pain. At the upazilla health complex, the attending doctors conducted a thorough examination, administered tetanus toxoid, and referred the patient to the district hospital for further treatment. After an approximate 8-h journey, the patient arrived at Sadar Hospital (district hospital) where the initial dose (10 vials) of nonspecific polyvalent anti-venom was administered. Concurrently, wound care and third-generation cephalosporin were initiated. Upon examination, the attending doctors noted the patient's agitation, icteric appearance, and extensive swelling in the bitten leg, extending up to the mid-thigh (Fig. 3). Since there was no significant improvement observed in the patient's overall condition, a second anti-venom treatment (10 vials) was administered. Considering the lack of progress, the medical team recommended admission to Dhaka Medical College Hospital. Upon admission, the patient received symptomatic management, and a third dose of anti-venom (10 vials) was administered.

During admission, laboratory investigations showed a hemoglobin level of 12.7 g/dl, a total leukocyte count of 33,300/mm^3^ with a normal differential count, and a platelet count of 6000/mm^3^. Both serum creatinine and urea were high, at 6.09 mg/dl and 160 mg/dl, respectively. In addition, fibrinogen was 257 mg/dl, procalcitonin was > 200, D-dimer level was 17.38, FDP was 109.48 and serum CPK was 3999 U/L. His troponin level was 0.12 ng/dl, INR was 1.2, APTT was 30.9 and PT was 14.0. Serum bilirubin was found to be 9.30 mg/dl, with the predominant indirect bilirubin measuring 6.4 mg/dl, LDH 518 U/L and serum alkaline phosphate 46.7 U/L. level of serum albumin was 2.7 g/dl. The patient was icteric, semiconscious, and had very low urine output. Due to the rapid development of coagulopathy, decline in renal function, and multiorgan involvement, the patient was shifted to the ICU and started on dialysis. Initially, improvement was very slow, and at 08 days after the bite, patients developed hematuria and pancytopenia with respiratory distress, and endotracheal intubation was performed based on immediate action along with other standard care.

He received a total of four doses (40 vials) of antivenom and underwent seven sessions of dialysis, 6 units of fresh frozen plasma, 5 units of platelet transfusions and 3 units of whole blood transfusion. After seven days of mechanical ventilation and aggressive treatment, the patient's laboratory reports returned to normal, and he became clinically stable. After 26 days of hospitalization, he was discharged with advice to home with no residual disabilities (Fig. 2**)**.

#### Outcome of the patient

The patient survived with no residual disabilities. Now he regularly took follow up over the telephone and take necessary advice on regular fashion.

#### Outcome of the snake

Russell viper snake is also known as the 'Guiyaporaa snake' in the local language. The snake was not harmed and allowed to escape. The patient and their companion accurately identified the snake. After admission, a different snake sample was presented to them, and it was correctly recognized, thus confirming the presence of a Russell viper.

## Discussion

The two clinical cases presented here illustrate the public health challenges encountered in the diagnosis, treatment, and management of snakebite victims in Bangladesh.

In both cases, the patients presented late, 18 and 58 h after the bite, respectively. This delay can be attributed to several factors, including limited healthcare infrastructure in rural areas, lack of awareness about the snakebites, and dependance on ojhas. The injurious treatments and the inability to identify the envenomation by the ojhas led to life threatening complications such as acute renal failure, hemolysis, and coagulopathy. In a previous national community-based health and injury survey, 61% of victims were reported seeking immediate care from these ojhas as pre hospital treatment [3]. In addition, using various medicinal herbs such as thrones of flowers or chilies in a fresh wound may raise the infection risks, much like in our case reports. Previously, 35 different species of medicinal herbs were reported to be used by ojhas in the southwest region of Bangladesh to treat snake bites [4].

In many rural areas where modern healthcare facilities are limited, ojhas are often the only healthcare providers available to local communities, and they can be reached quickly and easily. Numerous countries are now integrating traditional healers with their primary health care including countries like Ghana, Zimbabwe, Malawi etc. Similar to the approach in Nepal, it would be beneficial for all traditional healers to be registered accordingly in health care system of Bangladesh [5]. They should be facilitated by structured hands-on training on snake identification, provision of first aid, and understanding of the referral system. Zimbabwe has already successfully incorporated their traditional healers in combating TB and HIV [6]. Previous research conducted in Ghana highlighted a willingness among traditional practitioners to receive training and engage in collaborative efforts in managing snakebite cases [7]. Some earlier studies have already confirmed the effectiveness of combining traditional healers into the snake bite cases [8].

Furthermore, Limited access to healthcare in rural areas poses challenges in snakebite management. Both patients received partial treatment at district hospitals and were referred to tertiary care centers. Reduced number of well-equipped hospitals and expert physicians contributes to delayed and suboptimal care. Physicians in snake bite prone area should be well trained with the syndromic approach of snake bite and adhere to the national guideline of Bangladesh. In addition, establishing dedicated snakebite wards and ICUs can ensure timely and appropriate administration of antivenom and comprehensive care, improving patient outcomes. Upgrading health infrastructures with availability of antivenom, initial resuscitation support and including ambulance services will lead to better patient outcome.

Both patients developed multiple complications such as coagulopathy, renal failure, and multiorgan involvement, consistent with the systemic effects of snakebites, notably from Russell's vipers [9, 10]. Coagulopathy and renal failure are known outcomes of venomous bites, underscoring the need for close renal and coagulation monitoring [11]. A study from Myanmar found that the key predictor for the development of acute kidney injury is the time interval between the bite and administration of anti-snake venom. They also reported that early injection of anti-snake venom is associated with reduced events of coagulopathy [12]. Delayed administration of antivenom, in both cases, led to these severe complications. The management approach involved critical care, hematology, and nephrology specialists, emphasizing the importance of a multidisciplinary approach.

Our case report also highlights the significant economic burden of snakebite management in Bangladesh. Transportation costs for victims in rural areas to tertiary health facilities are substantial. Once at the tertiary care centers, expenses continue to accumulate, including ICU stays, intravenous fluids, treatment of complications, and supportive measures. This financial strain is particularly high in a country like Bangladesh. Managing a venomous snakebite case costs around US$231, nearly seven times more than a non-venomous snakebite case, as revealed by a previous study [13].

To summarize, our findings highlight the importance of early recognition and management of complications in patients with Russell’s viper envenomation. Overall, addressing these factors through improved education, healthcare infrastructure, and public health initiatives could help to reduce the mortality rate by snake bite. Educating high-risk populations about the importance of seeking hospital care promptly, instead of initially reaching to traditional healers, can lead to effective treatment outcomes. Finally, ensuring that anti-venom is readily accessible in remote areas is crucial to improve survival rates for snakebite victims. These strategies combined can significantly reduce their associated morbidity and mortality of snake bite cases.

## Conclusion

In conclusion, our case series illustrates the importance of prompt and appropriate medical management of snakebites to improve patient outcomes in rural areas of Bangladesh. They underscore the need for education and training of traditional healers on the proper management of snakebites, as well as the need for improved access to medical facilities capable of providing appropriate care. Finally, it is high time to adopt a collaborative and multidisciplinary approach involving government authorities, healthcare professionals, researchers, and non-governmental organizations to implement these strategies successfully.

## Data Availability

All data were available to the lead author and could be found upon the reasonable request to the corresponding author.

## Abbreviations

APTT: Activated partial thromboplastin time
Ci: Chloride
CPK: Creatine phosphor kinase
CXR: Chest X ray
Echo: Echocardiogram
FDP: Fibrinogen degradation product
FFP: Fresh frozen plasma
ICU: Intensive care unit
LDH: Lactate dehydrogenase
N^a^: Sodium
PT: Prothrombin time
RBS: Random blood sugar
USG: Ultrasonogram
WHO: World Health Organization

## References

[1] Chippaux JP (2017). Snakebite envenomation turns again into a neglected tropical disease!. J Venom Anim Toxins Including Trop Dis.

[2] Ahsan F, Saeed A (2018). Russell’s Viper (Daboia Russelii) in Bangladesh: its boom and threat to human life. J Asiat Soc Bangladesh Sci..

[3] Hossain J, Biswas A, Rahman F, Mashreky SR, Dalal K, Rahman A (2016). Snakebite epidemiology in Bangladesh—a national community based health and injury survey. Health.

[4] Hasan MN, Azam NK, Ahmed MN, Hirashima A (2016). A randomized ethnomedicinal survey of snakebite treatment in southwestern parts of Bangladesh. J Tradit Complement Med.

[5] Subedi B. Perspective chapter: integrating traditional healers into the National Health Care System—a review and reflection. In: Rural health—investment, research and implications. IntechOpen; 2023. https://www.intechopen.com/chapters/85900.

[6] Madamombe I (2006). Traditional healers boost primary health care. Afr Renew.

[7] Steinhorst J, Aglanu LM, Ravensbergen SJ, Dari CD, Abass KM, Mireku SO (2021). ‘The medicine is not for sale’: practices of traditional healers in snakebite envenoming in Ghana. PLoS Negl Trop Dis..

[8] Steinhorst J, Tianyi FL, Habib AG, Oluoch GO, Lalloo DG, Stienstra Y (2022). Uniting behind a common goal: collaboration between traditional healers and allopathic health care workers to improve rural snakebite care. Toxicon X..

[9] Siddique MAB, Rahman MM, Kabir HAKM, Mallik MU, Habibullah M, Hassan MM (2021). Experience of managing snake bite cases in a medicine unit of tertiary care hospital in Bangladesh—a case series. J Med (Bangladesh).

[10] Banerjee R (2022). Epidemiology and clinical features of snake bite induced acute kidney injury patients in last decade and its longterm outcome—a single center experience. Indian J Appl Res.

[11] Hung DZ, Yu YJ, Hsu CL, Lin TJ (2006). Antivenom treatment and renal dysfunction in Russell’s viper snakebite in Taiwan: a case series. Trans R Soc Trop Med Hyg.

[12] Alfred S, Bates D, White J, Mahmood MA, Warrell DA, Thwin KT (2019). Acute kidney injury following Eastern Russell's Viper (Daboia siamensis) snakebite in Myanmar. Kidney Int Rep.

[13] Hasan SMK, Basher A, Molla AA, Sultana NK, Faiz MA (2012). The impact of snake bite on household economy in Bangladesh. Trop Doct.

